# *Momordica charantia* Attenuates Hepatic Steatosis in High‐Fat, High‐Sucrose Diet‐Induced Obese Mice

**DOI:** 10.1155/ancp/2235628

**Published:** 2026-10-26

**Authors:** Hyun-Min Woo, Se-Yeong Park, Young-Hee Jo, Sung-Jin Lee, Soo Ho Yeon, Hee-Soo Han, Tae Hoo Yi, Yeon Ju Kim, Yong-Mu Kim, Hyocheol Bae

**Affiliations:** ^1^ Department of Convergent Biotechnology and Advanced Materials Science College of Life Sciences Kyung Hee University Yongin 17104 Republic of Korea khu.ac.kr; ^2^ Ingredient R&D Center Kolmar BNH Co., Ltd., 61 Heolleung-ro 8-gil, Seocho-gu Seoul 06800 Republic of Korea

**Keywords:** cucurbitaceae, hepatic steatosis, high-fat, high-sucrose diet, lipid metabolism, *Momordica charantia L*., obesity

## Abstract

High‐fat, high‐sucrose (HFHS) diet consumption is a major contributor to obesity and liver‐related metabolic disorders. *Momordica charantia* ethanolic extract powder (MC), derived from a traditional climbing plant, has been shown to exert multiple biological activities, including exhibiting antidiabetic, antioxidant, and antiinflammatory activities. Building on these properties, the present study investigated the potential effects of MC on hepatic lipid metabolism and obesity‐related parameters in mice fed a HFHS diet. Male C57BL/6J mice (6 weeks old) were fed an HFHS diet for 4 weeks and subsequently divided into four dietary groups, receiving either continued HFHS, *Cissus quadrangularis* (CQ; positive control), MC 125 (125 mg/kg), or MC 250 (250 mg/kg) for an additional 12 weeks. Compared with the HFHS group, MC supplementation was associated with lower body weight gain, hepatic lipid accumulation, and epididymal adipocyte size. Histological findings were consistent with these changes in adipose tissue. At the molecular level, MC supplementation was associated with altered hepatic expression of genes involved in lipid metabolism, including decreased expression of the lipogenesis‐related genes *SREBP-1c*, *ACC*, *FAS*, and *SCD1*, as well as alterations in *C/EBPα* and *PPARγ* expression. In addition, the expression of fibrosis‐related genes *Col1α1* and *αSMA* was decreased in the MC‐supplemented groups. These findings suggest that MC supplementation may modulate hepatic lipid metabolism‐ and fibrosis‐related gene expression in HFHS diet‐fed mice. Collectively, the present findings suggest that MC may influence metabolic responses associated with chronic consumption of a HFHS diet.

## 1. Introduction

Obesity and overweight are global health challenges and the subject of extensive research. Obesity is a major contributor to chronic and progressive diseases, such as diabetes, and is shaped by a variety of lifestyle‐related factors, particularly dietary patterns and levels of physical activity. Its prevalence has made it the leading nutritional disorder in the Western world [1, 2]. Metabolic imbalance, resulting from increased caloric intake and decreased energy expenditure, leads to obesity, particularly when diets are high in fat, calories, and fructose. Over the past 40 years, fructose consumption has increased by more than 30%, paralleling the rise in overweight, obesity, and metabolic syndrome. Fructose reduces satiety, stimulates food intake, and alters lipid and carbohydrate metabolism, thereby promoting fat synthesis [3, 4]. Excess energy is primarily stored in adipose tissue but can also accumulate in organs such as the liver, contributing to organ dysfunction [5]. Adipose tissue plays a central role in maintaining energy homeostasis by buffering energy surges after food intake. However, when its buffering capacity is exceeded, abnormal lipid accumulation can cause metabolic damage in other tissues, including the liver and skeletal muscle [6]. This process closely links obesity to the pathogenesis of metabolic diseases.

To prevent and manage overweight, obesity, and related noncommunicable diseases, individuals adopt healthy dietary patterns, increased physical activity, and lifestyle modifications, such as the Mediterranean diet, low‐carbohydrate diets, and intermittent fasting. Dietary supplements and pharmacological interventions are also widely used. While many supplements are considered safe, indiscriminate use can lead to toxic reactions or drug interactions [7]. Consequently, there is a growing interest in developing functional foods from plant extracts that minimize side effects and support metabolic health.

*Momordica charantia* belongs to the *Cucurbitaceae* family and grows as a climbing vine across tropical regions such as Asia, the Amazon basin, East Africa, and the Caribbean. It has a long history of medicinal use, particularly in the prevention and treatment of diabetes, in many developing countries [8–10]. Studies have demonstrated its immunomodulatory, antidiabetic, antiviral, antibacterial, antioxidant, antiulcer, and antiinflammatory properties [9]. Recent research has highlighted its potential role in modulating insulin‐related responses and pancreatic β‐cell function. There is also evidence that the *Momordica charantia* extract may attenuate high‐fat diet‐induced disruption of the blood‐brain barrier and inflammatory cytokine expression, as well as affect insulin sensitivity and pancreatic function [11–13].

However, most existing studies have focused on its neuroprotective and antiinflammatory effects or on its impact on insulin regulation, obesity, and diabetes. There is a relative paucity of research concerning the effects of *Momordica charantia* on liver health, particularly regarding hepatic fat accumulation. Therefore, in this study, we refer to unripe *Momordica charantia* ethanolic extract powder as MC and evaluate its effects on obesity‐related parameters, hepatic lipid accumulation, and adipocyte morphology in vivo. This study aims to investigate the effects of MC on lipid metabolism‐related parameters under high‐fat, high‐sucrose (HFHS) diet conditions, providing experimental evidence for its potential application as a functional food ingredient.

## 2. Materials and Methods

### 2.1. Preparation of *Momordica charantia*

The unripe MC (Sugar Katcher S52) was prepared by extracting unripe *Momordica charantia* with 70% ethanol at 70°C for 6 h. The extract was then spray‐dried to obtain a powder. The extract powder used in this study was provided by KolmarBNH Co., Ltd. (Sejong, South Korea). According to the manufacturer, the native drug‐to‐extract ratio (DER genuine) is 3.26:1 (w/w), meaning that 3.26 g of starting material was used to produce 1 g of native extract. This ingredient was approved as a Health Functional Food Ingredient by the Korean Ministry of Food and Drug Safety in 2021 (Approval Number 2020‐14) and is standardized to contain 1.08 mg/g of GABA (*γ*‐aminobutyric acid).

### 2.2. Phytochemical Characterization

The GABA content was analyzed by KolmarBNH Co., Ltd. (Sejong, South Korea) using their in‐house validated method with an automatic amino acid analyzer (LA8080, Hitachi High‐Tech Corp.). Amino acid analysis was conducted using an ion‐exchange column (Number 2622SC‐PF, 4.6 × 60 mm; Hitachi Ltd.) maintained at 30–70°C, equipped with a postcolumn ninhydrin derivatization system (reactor temperature: 135°C). Sodium‐citrate buffer solutions (B1–B6; PF‐SET, Kanto Chemical, Japan) were delivered by pump 1 at 0.35 mL/min and ninhydrin coloring solution (R1–R2; Wako Pure Chemical Industries, Japan) by pump 2 at 0.30 mL/min. Detection was performed at 570 nm using a visible/UV detector. The total runtime was 157.3 min. Representative chromatograms showing GABA standard solution and GABA analysis in the MC (test solution) are presented in Figure S1 (manufacturer’s CoA).

### 2.3. Quality Control and Batch Information

All experiments in this study were performed using a single batch of extract (Batch Number 20230207‐P1/R23110729). According to the manufacturer’s documentation, the batch complied with predefined specifications for appearance, foreign matter, indicator compound analysis and content, microbial purity, heavy metals, and residual solvents. Batch‐to‐batch consistency was monitored by routine comparison of chromatographic fingerprints and marker compound content against an in‐house reference standard.

### 2.4. Animal Model and Experimental Design

All animal procedures conducted in this study received approval from the Institutional Animal Care and Use Committee of Kyung Hee University, Yongin‐si, Gyeonggi‐do, Korea (KHGASP‐24‐690) in March 2024 and were performed in accordance with institutional guidelines. 6‐week‐old C57BL/6 male mice (*n* = 6 per group) were purchased from RAONBIO Co., Ltd. (Yongin, Gyeonggi‐do, Korea). Mice were housed under controlled conditions (25 ± 3°C, 53 ± 8% humidity, 12 h light/dark cycle, and 10–12 air changes per hour). After a 1‐week acclimatization period, mice were randomly assigned to five groups (*n* = 6 per group): normal diet (NC), HFHS diet, HFHS + *Cissus quadrangularis* extract (HFHS + CQ, 60 mg/kg), HFHS + low‐dose unripe *Momordica charantia* ethanol extract (HFHS + MC 125 mg/kg), and HFHS + high‐dose unripe *Momordica charantia* ethanol extract (HFHS + MC 250 mg/kg) (Table 1).

**Table 1:** Composition of the experimental diet.

Groups	Diet composition
NC	Normal diet
HFHS	High‐fat, high‐sucrose diet
CQ	High‐fat, high‐sucrose diet + *Cissus quadrangularis*
MC 125	High‐fat, high‐sucrose diet + *Momordica charantia* L 125 mg/kg
MC 250	High‐fat, high‐sucrose diet + *Momordica charantia* L 250 mg/kg

All groups except the NC group were fed a HFHS diet for 4 weeks to induce obesity. After this induction period, the respective diets and extract treatments were continued for an additional 12 weeks according to the group assignment. Mice were fasted for at least 12 h before euthanasia by CO_2_ inhalation. Following collection, liver and white adipose tissues (epididymal and retroperitoneal fat) were rinsed with phosphate‐buffered saline and weighed. Samples were then allocated either for fixation in 4% paraformaldehyde or for storage at −80°C before qRT‐PCR analysis.

### 2.5. Body Weight, Food, and Water Intake Measurements

From the first day of administration, the body weight was measured once a week at the same time. Food intake was determined by measuring the remaining food and the newly supplied food and then calculating the difference to obtain the total intake. Water intake was recorded using the same method (Figure 1).

**Figure 1:**
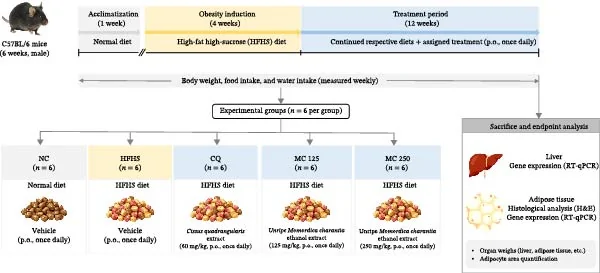
Schematic diagram of animal experiment. Mice underwent a 1‐week acclimatization period, 4 weeks of obesity induction, and a 12‐week treatment period. Body weight, food intake, and water intake were monitored weekly, followed by endpoint analyses.

### 2.6. Quantitative Real‐Time Polymerase Chain Reaction (qRT‐PCR) Analysis

Liver and adipose tissues were homogenized, and total RNA was extracted using the TRIzol reagent (Thermo Fisher Scientific, Waltham, MA, USA) according to the manufacturer’s instructions. Chloroform was added, and samples were incubated for 10 min, followed by centrifugation at 13,000 rpm for 10 min at 4°C. The supernatant was collected, and RNA purity and concentration were measured using a NanoDrop spectrophotometer. cDNA was synthesized from total RNA using the PrimeScript First‐Strand cDNA Synthesis Kit (TaKaRa Bio Inc., Kusatsu, Japan). qRT‐PCR was performed using a QuantStudio 3 system (Stratagene, San Diego, CA, USA). Primer sequences are listed in Table 2. Target mRNA expression was normalized to *β*‐actin.

**Table 2:** List of primer sequences used in this study.

Gene name	Primer sequence (gene accession number: position)
*PPARγ*	Forward: 5′–TCGCTGATGCACTGCCTATG–3′ (NM 011146: 115–134) Reverse: 5′–GAGAGGTCCACAGAGCTGATT ‐3′ (NM 011146: 183–203)
*Col1α1*	Forward: 5′–TCCGGCTCCTGCTCCTCTTA–3′ (NM_007742: 165–184) Reverse: 5′–GTATGCAGCTGACTTCAGGGATGT ‐3′ (NM_007742: 219–242)
*αSMA*	Forward: 5′–ACTCTCTTCCAGCCATCTTT–3′ (NM_007392: 891–910) Reverse: 5′–ATAGGTGGTTTCGTGGATGC ‐3′ (NM_007392: 931–950)
*C/EBPα*	Forward: 5′–CAAGAACAGCAACGAGTACCG–3′ (NM_007678: 966–986) Reverse: 5′–GTCACTGGTCAACTCCAGCAC ‐3′ (NM_007678: 1069–1089)
*SCD1*	Forward: 5′–GCCCCTGCGGATCTTCCTTA–3′ (NM_009127: 684–703) Reverse: 5′–GAATTGTGAGGGTCGGCGTG ‐3′ (NM_009127: 787–806)
*SREBP-1 c*	Forward: 5′–TGTGGAGCTCAAAGACCTG–3′ (NM_001358315: 979–997) Reverse: 5′–TGTGCTGCAAGAAGCGGATG ‐3′ (NM_001358315: 1057–1076)
*ACC*	Forward: 5′–GAATCTCCTGGTGACAATGC–3′ (NM_133360: 3277–3296) Reverse: 5′–GGTCTTGCTGAGTTGGGTTA ‐3′ (NM_133360: 3363–3382)
*FAS*	Forward: 5′–CTGAGATCCCAGCACTTCTT–3′ (NM_007988: 1618–1637) Reverse: 5′–GCCTCCGAAGCCAAATGAG ‐3′ (NM_007988: 1700–1717)

Abbreviations: ACC, acetyl‐CoA carboxylase; C/EBPα, CCAAT/enhancer‐binding protein alpha; Col1α1, collagen type Ⅰ alpha 1; FAS, fatty acid synthase; PPARγ, peroxisome proliferator‐activated receptor gamma; SCD1, stearoyl‐coenzyme a desaturase 1; SREBP‐1c, sterol regulatory element binding protein‐1c; αSMA, alpha‐smooth muscle actin.

### 2.7. Hematoxylin and Eosin (H&E) Staining of Epididymal Adipose Tissue

Epididymal adipose tissue samples were fixed in 4% paraformaldehyde and stained with H&E by CHAON (Yongin‐si, Gyeonggi‐do, Korea). Adipocyte size was evaluated using an Olympus microscope (Tokyo, Japan) on a 20 μm scale. The adipocyte area was measured using ImageJ software (LOCI, University of Wisconsin, USA) and expressed as the absolute adipocyte area (μm^2^). For comparative analysis, the mean adipocyte area of the HFHS group was set to 100%, and adipocyte sizes in the CQ, MC 125, and MC 250 groups were expressed as relative percentages of the HFHS group. For each group, adipocyte areas were quantified from randomly selected microscopic fields, and the mean adipocyte area was calculated for statistical analysis.

### 2.8. Immunohistochemistry (IHC)

Immunohistochemical staining was performed using the VECTASTAIN ABC Kit (Vector Laboratories, CA, USA) to evaluate the expression of PPARγ and C/EBPα in the liver tissue and PPARγ expression in the adipose tissue. Paraffin‐embedded tissues were sectioned, deparaffinized in xylene, and rehydrated through a graded ethanol series (100%–70%). Antigen retrieval was performed using citrate buffer, followed by incubation in 3.3% hydrogen peroxide to block the endogenous peroxidase activity. After blocking with the blocking solution, the sections were incubated overnight at 4°C with primary antibodies against PPARγ (1:400) and C/EBPα (1:200). The sections were then washed twice with PBS and incubated with HRP‐conjugated secondary antibodies for 1 h at 37°C. Following PBS washes, the VECTASTAIN ABC reagent was applied according to the manufacturer’s instructions, and immunoreactivity was visualized using a DAB substrate solution. The sections were subsequently washed with PBS, dehydrated through graded ethanol, cleared in xylene, and mounted. Tissue morphology and immunostaining were observed using a microscope‐mounted digital camera system (E3ISPM03100KPA, ToupTek Photonics Co., Ltd., Hangzhou, China).

### 2.9. Statistical Analysis

Statistical analysis was performed using GraphPad Prism 7. Data are presented as the mean ± standard deviation (SD). One‐way ANOVA, followed by Tukey’s posthoc test, was used for between‐group comparisons. Body weight data were analyzed using two‐way repeated‐measures ANOVA, followed by Dunnett’s multiple comparisons test for comparisons between the HFHS group and the other groups at each time point. A *p*‐value of <0.05 was considered statistically significant.

## 3. Results and Discussion

After 12 weeks of dietary intervention, the HFHS group showed a significant increase in body weight compared with the NC group (Figure 2). In contrast, the MC‐supplemented groups showed numerically lower body weight gain than the HFHS group, with reductions of 1.76% in MC 125 and 3.33% in MC 250; however, these differences were not statistically significant. Food and water intake were also lower in MC‐treated groups than in HFHS controls (Table 3); food intake decreased by 11.66% in MC 125 and 44.27% in MC 250, while water intake decreased by 19.33% and 28.89%, respectively. Although food intake was lower in the MC 250 group than in the HFHS group, the food efficiency ratio (FER) was comparable between the two groups (Table S1). These findings suggest that differences in food intake should be considered when interpreting the lower body weight observed in the MC 250 group. Because excess dietary carbohydrates and fructose can provide sustained substrate flux to the liver and thereby promote lipogenesis and weight gain [14–16], reduced energy intake may have partially contributed to the improved body‐weight phenotype. Accordingly, the present findings indicate that MC reduced HFHS‐associated weight gain through mechanisms that may extend beyond simple reductions in food intake, although follow‐up studies under energy intake‐controlled (intake‐matched) conditions will be required to clarify the extent of MC’s intake‐independent metabolic effects.

**Figure 2:**
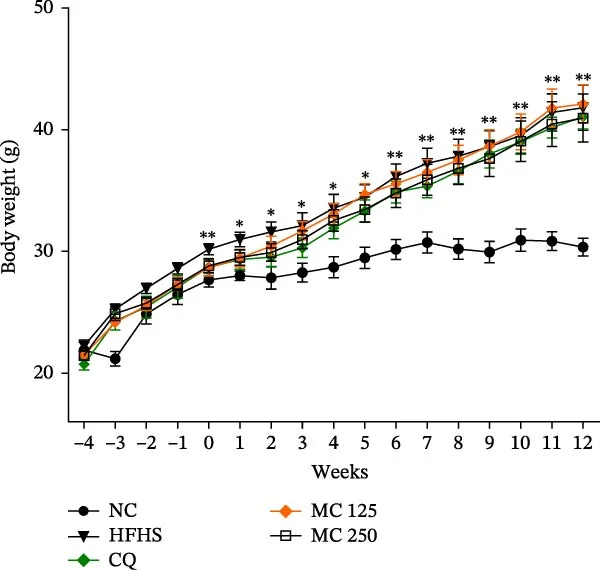
Changes in body weight in each group from obesity induction through the 12‐week intervention period. Six mice were included in each group, and data are presented as the mean ± standard error of the mean (SEM). Statistical significance was assessed using two‐way repeated‐measures ANOVA followed by Dunnett’s multiple comparisons test at each time point. Asterisks indicate significant differences between the HFHS and NC groups during the 12‐week intervention period ( ^∗^ *p* < 0.05 and  ^∗∗^ *p* < 0.01). No significant differences were observed between the HFHS group and the CQ, MC 125, or MC 250 groups at any time point during the intervention period ( ^∗^ *p* > 0.05). *Note:* NC, normal diet; HFHS, high‐fat, high‐sucrose diet; CQ, high‐fat, high‐sucrose diet supplemented with *Cissus quadrangularis* extract powder; MC 125, high‐fat, high‐sucrose diet supplemented with unripe *Momordica charantia* ethanolic extract powder at 125 mg/kg; MC 250, high‐fat, high‐sucrose diet supplemented with unripe *Momordica charantia* ethanolic extract powder at 250 mg/kg.

**Table 3:** Food and water intakes in all experimental groups.

Parameter	NC	HFHS	CQ	MC 125	MC 250
Food intake (g/week/mouse)	3.81 ± 0.159	4.63 ± 0.351	3.54 ± 0.164	4.09 ± 0.186	2.58 ± 0.079
Drink intake (mL/week/mouse)	8.44 ± 0.701	4.81 ± 0.325	5.14 ± 0.707	3.88 ± 0.129	3.42 ± 0.087

Hepatic lipogenesis was assessed by analyzing the expression of *SREBP-1c*, a key transcription factor, and its downstream target genes (*SCD1*, *FAS*, and *ACC*) via qRT‐PCR. The HFHS group exhibited approximately a twofold increase in *SREBP-1c* expression compared with the NC group. Notably, the MC 125 and MC 250 groups showed a significant reduction in *SREBP-1c* expression, decreasing by 46.66% and 59.10%, respectively, relative to the HFHS group (Figure 3A). *FAS*, an enzyme crucial for de novo fatty acid synthesis and a downstream target of *SREBP-1c*, displayed a dose‐dependent decrease in the MC groups, with reductions of 67.95% and 75.15%, respectively, compared with the HFHS group. These reductions were greater than those observed in the CQ group (Figure 3B). Similarly, *ACC*, involved in the early stages of fatty acid biosynthesis, showed dose‐dependent reduction of 51.48% and 65.17% in the MC‐treated groups (Figure 3C). *SCD1*, a key regulator of lipogenesis, exhibited marked reductions in expression, with expression decreasing by 82.78% and 89.32% in the MC 125 and MC 250 groups, respectively, compared with that in the HFHS group (Figure 3D). These coordinated transcriptional changes align with the established role of *SREBP-1c* as a central lipogenic transcription factor induced by excessive fructose intake [14–18]. Upon activation, *SREBP-1c* upregulates enzymes that drive de novo lipogenesis and triglyceride synthesis (*ACC*, *FAS*, and *SCD1*), thereby promoting hepatic lipid accumulation [19–22]. Therefore, the lower expression of *SREBP-1c* and its related lipogenic genes in the MC‐supplemented groups suggests that MC supplementation was associated with alterations in hepatic lipogenesis‐related gene expression under HFHS feeding.

**Figure 3:**
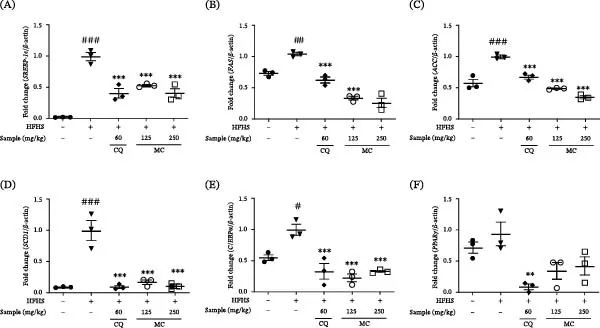
qRT‐PCR analysis of the expression levels of genes related to fatty acid synthesis, lipid synthesis, and storage in the liver (SREBP‐1c (A), FAS (B), ACC (C), SCD1 (D), C/EBPα (E), and PPARγ (F)). The data are shown as mean ± SD (*n* = 6). Statistical significance was indicated as #*p* < 0.05, ##*p* < 0.01, and ###*p* < 0.001 for differences between the NC and HFHS groups, and  ^∗^ *p* < 0.05,  ^∗∗^ *p* < 0.01, and  ^∗∗∗^ *p* < 0.001 for differences between the treatment groups and the HFHS group. *Note:* NC, normal diet; HFHS, mice fed a high‐fat, high‐sucrose diet; CQ, mice fed with a high‐fat, high‐sucrose diet supplemented with *Cissus quadrangularis* extract powder; MC 125, mice fed with a high‐fat, high‐sucrose diet supplemented with unripe *Momordica charantia* ethanolic extract powder at 125 mg/kg; MC 250, mice fed with a high‐fat, high‐sucrose diet supplemented with unripe *Momordica charantia* ethanolic extract powder at 250 mg/kg.

CCAAT/enhancer‐binding protein alpha (*C/EBPα*) and peroxisome proliferator‐activated receptor gamma (*PPARγ*) are key transcriptional factors involved in lipogenesis, lipid oxidation, and storage. To evaluate their expression in relation to hepatic lipid metabolism, qRT‐PCR analysis was performed. The results revealed that the *C/EBPα* expression in the HFHS group was ~34% higher than in the NC group. However, administration of MC 125 and MC 250 significantly reduced the *C/EBPα* expression by 77.07% and 66.91%, respectively. Similarly, *PPARγ* expression was reduced by 63.66% and 55% in the MC 125 and MC 250 groups, with reductions comparable to those observed in the CQ‐treated group (Figure 3E,F). Both *C/EBPα* and *PPARγ* are implicated in lipid metabolic regulation, and their induction is associated with enhanced lipid storage programs and TG accumulation [19, 23–25]. Accordingly, the decreased expression of these transcription factors in the MC‐supplemented groups was consistent with the changes observed in other hepatic lipid metabolism‐related genes [25, 26]. Notably, *PPARγ* is minimally expressed in the liver under normal physiological conditions but can become induced in steatotic states, where hepatocyte *PPARγ* upregulation has been linked to hepatic fat accumulation [27, 28]. Thus, the lower hepatic *PPARγ* expression observed following MC supplementation may be relevant to the differences in hepatic lipid accumulation observed among the experimental groups [29].

The progression of hepatic steatosis can lead to liver fibrosis, and collagen type I alpha 1 (*Col1α1*) and alpha‐smooth muscle actin (*αSMA*) can be major contributors to liver fibrosis when overexpressed [30–32]. The expression of *Col1α1* and *αSMA* was ~72% higher in the HFHS group than in the NC group. In the MC 125 and MC 250 groups, the expression of *Col1α1* was reduced by 47.58% and 44.09%, respectively, compared with that of the HFHS group. The expression of *αSMA* was also reduced by 45.22% and 60.98% in the MC 125 and MC 250 groups, respectively, compared with the HFHS group (Figure 4A,B). *Col1α1* contributes to extracellular matrix accumulation in fibrotic tissues, and *αSMA* is a marker of hepatic stellate cell activation, both commonly linked to fibrosis progression [33–35]. Therefore, the lower expression of Col1α1 and αSMA in the MC‐supplemented groups indicates changes in fibrosis‐related gene expression under HFHS diet conditions. However, these transcriptional findings alone are insufficient to establish an antifibrotic effect. Further histological and protein‐level validation is required. Future studies should identify the major bioactive components of MC and evaluate fibrosis‐related signaling pathways (e.g., TGF‐β/Smad and Wnt/β‐catenin) in hepatic stellate cells to further characterize these observations.

**Figure 4:**
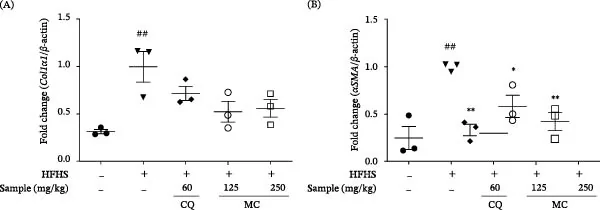
qRT‐PCR analysis of the mRNA expression levels of genes associated with liver fibrosis in the liver (*Col1α1* (A), *αSMA* (B)). Data are presented as mean ± SD (*n* = 6). Statistical significance was indicated as ##*p* < 0.01 for differences between the NC and HFHS groups, and  ^∗^ *p* < 0.05,  ^∗∗^ *p* < 0.01 for differences between the treatment groups and the HFHS group. *Note:* NC, normal diet; HFHS, mice fed with a high‐fat, high‐sucrose diet; CQ, mice fed with a high‐fat, high‐sucrose diet supplemented with *Cissus quadrangularis* extract powder; MC 125, mice fed with a high‐fat, high‐sucrose diet supplemented with Unripe *Momordica charantia* ethanolic extract powder 125 mg/kg; MC 250, mice fed with a high‐fat, high‐sucrose diet supplemented with Unripe *Momordica charantia* ethanolic extract powder 250 mg/kg.

H&E staining was performed to evaluate the morphological changes in the epididymal adipose tissue, and the results are shown in Figure 5. To analyze the area of adipocytes in the epididymal adipose tissue in NC, HFHS, CQ, MC 125, and MC 250 groups, the mean adipocyte area was quantified using ImageJ software and expressed as a percentage relative to the HFHS group, which was set to 100% (Figure 5F).

**Figure 5:**
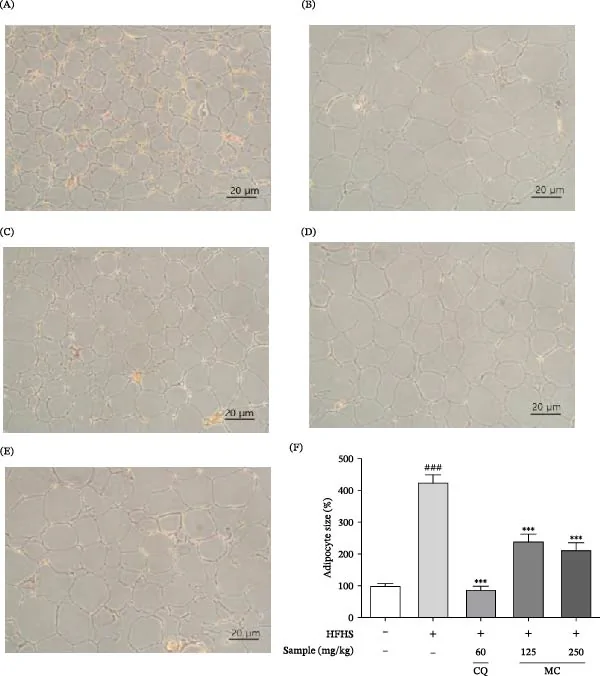
Histopathological analysis of epididymal adipose tissue and quantitative evaluation of adipocyte size. Representative H&E‐stained sections of epididymal adipose tissue from the NC (A), HFHS (B), CQ (C), MC 125 (D), and MC 250 (E) groups are shown at ×200 magnification (scale bar = 20 µm). Adipocyte area was quantified from the selected H&E‐stained images using ImageJ software. Quantitative analysis of adipocyte size is shown in (F) and expressed as a percentage relative to the HFHS group. Data are presented as the mean ± SD. Statistical significance was determined by comparing the CQ, MC 125, and MC 250 groups with the HFHS group. Asterisks indicate significant differences ( ^∗^ *p* < 0.05,  ^∗∗^ *p* < 0.01, and  ^∗∗∗^ *p* < 0.001). *Note:* NC, normal diet; HFHS, mice fed a high‐fat, high‐sucrose diet; CQ, mice fed a high‐fat, high‐sucrose diet supplemented with *Cissus quadrangularis* extract powder; MC 125, mice fed a high‐fat, high‐sucrose diet supplemented with unripe *Momordica charantia* ethanolic extract powder at 125 mg/kg; MC 250, mice fed a high‐fat, high‐sucrose diet supplemented with unripe *Momordica charantia* ethanolic extract powder at 250 mg/kg.

As shown in Figure 5, adipocytes in the epididymal adipose tissue of the NC group were relatively small and closely arranged. In contrast, the HFHS group exhibited enlarged adipocytes compared with those of the NC group. In the MC‐supplemented groups, the mean adipocyte area was lower than that in the HFHS group, with a greater reduction observed in the MC 250 group than in the MC 125 group. Adipose tissue expansion and adipocyte hypertrophy are strongly associated with obesity and systemic metabolic complications [36]. Adipocyte enlargement typically reflects an imbalance between lipid deposition and lipolysis [37], and saturation of adipose storage capacity can promote ectopic lipid deposition in organs such as the liver, muscle, and pancreas, contributing to metabolic dysfunction [38, 39]. Thus, the observed differences in adipocyte size following MC supplementation suggest that MC may be associated with alterations in adipose tissue morphology under HFHS diet conditions.

*SREBP-1c* and *PPARγ* play a central role in lipid metabolism not only in the liver but also in the adipose tissue. Accordingly, the expression of SREBP‐1c and PPARγ was evaluated in the adipose tissue, revealing an ~68.25% increase in the HFHS group compared with the NC group. The expression of *PPARγ* was reduced by 53.85% and 45.46% in the MC 125 and MC 250 groups, respectively, and the expression of *SREBP-1c* was also reduced by 63.06% and 56.41%, respectively, compared with that of the HFHS group (Figure 6). Mechanistically, *SREBP-1c* activates genes required for adipogenesis and lipogenesis, while *PPARγ* induces proteins involved in fatty acid uptake, binding, and transport; these transcription factors function in complementary ways to regulate adipogenesis and lipid accumulation in the adipose tissue [27, 40, 41]. Because adipogenic regulation is mediated by interconnected and complementary signaling interactions rather than a single linear pathway, the lower expression of adipose *SREBP-1c* and *PPARγ* in the MC‐supplemented groups suggests alterations in transcriptional regulation related to adipogenesis and lipid metabolism. Together with the observed differences in adipocyte size, these findings indicate consistent changes in lipid metabolism‐related parameters in the adipose tissue following MC supplementation.

**Figure 6:**
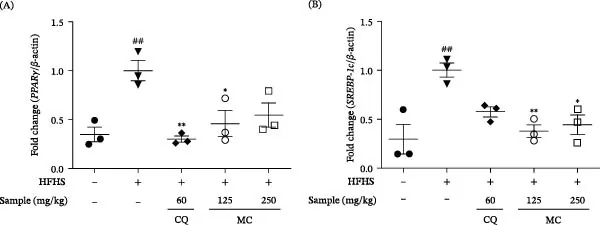
qRT‐PCR analysis of the expression levels of genes related to lipid metabolism in adipose tissue (*PPARγ* (A) and *SREBP-1c* (B)). The data are shown as mean ± SD (*n* = 6). Statistical significance was indicated as ##*p* < 0.01 for differences between the NC and HFHS groups, and  ^∗^ *p* < 0.05,  ^∗∗^ *p* < 0.01 for differences between the treatment groups and the HFHS group. *Note:* NC, normal diet; HFHS, mice fed a high‐fat, high‐sucrose diet; CQ, mice fed with a high‐fat, high‐sucrose diet supplemented with *Cissus quadrangularis* extract powder; MC 125, mice fed with a high‐fat, high‐sucrose diet supplemented with Unripe *Momordica charantia* ethanolic extract powder 125 mg/kg; MC 250, mice fed with a high‐fat, (high‐sucrose diet supplemented with Unripe *Momordica charantia* ethanolic extract powder 250 mg/kg).

Immunohistochemical staining was performed to evaluate the hepatic expression of PPARγ and C/EBPα. Although slight differences in DAB staining intensity were observed among the experimental groups, no distinct group‐dependent changes consistent with the RT‐qPCR results were identified. Likewise, PPARγ immunostaining in adipose tissue did not reveal apparent differences compared with the corresponding H&E findings, and no obvious changes in staining intensity were observed among the groups (Figure S2). These findings indicate that the transcriptional changes detected by RT‐qPCR were not clearly reflected by immunohistochemical staining. This discrepancy may be attributable to the limited sensitivity of qualitative IHC analysis and background staining, which may have reduced the ability to distinguish subtle differences in protein expression among the experimental groups. Therefore, further quantitative protein‐level analyses will be required to validate these findings.

Figure 7 summarizes the observed changes associated with MC supplementation under HFHS diet conditions, including changes in hepatic de novo lipogenesis‐related genes (*SREBP-1c*, *ACC*, *FAS*, and *SCD1*) and lipid storage‐related transcription factors (*C/EBPα* and *PPARγ*), together with changes in the adipose tissue. However, because MC supplementation was accompanied by reduced energy intake, particularly at the higher dose, the observed molecular and phenotypic changes should be interpreted in the context of intake effects. GABA was quantified in MC as a marker compound for quality control and extract standardization [42, 43]. However, the quantified GABA content alone does not indicate that GABA is the sole bioactive constituent responsible for the observed effects. *Momordica charantia* contains various phytochemicals, including triterpenoids, charantin, phenolic compounds, and flavonoids, which may contribute to its reported biological activities. Therefore, the changes observed in the present study cannot be attributed to a specific constituent based on the current data. Overall, MC supplementation was associated with changes in body weight and lipid metabolism‐related gene expression in HFHS‐fed mice. These observations warrant confirmation in follow‐up studies that control energy intake and further characterize the active constituents and underlying mechanisms.

**Figure 7:**
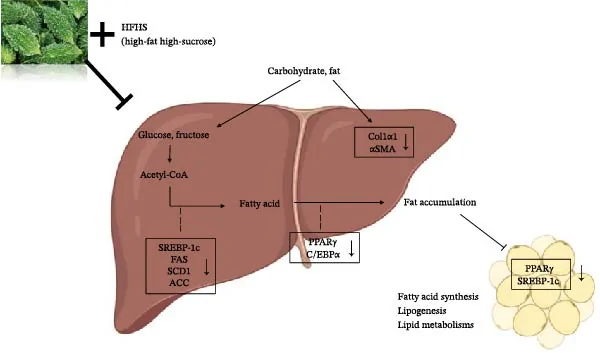
Schematic summary of the changes in lipid metabolism‐ and fibrosis‐related gene expression associated with MC supplementation under HFHS diet conditions.

## 4. Conclusion

Abnormal accumulation of fat in the liver contributes to obesity and increases the risk of metabolic complications, a condition exacerbated by the increasing consumption of high‐fat and high‐fructose diets. Hepatic fat accumulation is achieved by a variety of mechanisms, including delayed lipid excretion, increased lipid intake, increased lipid synthesis, or decreased fat oxidation [44]. To address these challenges, the present study investigated the effects of MC on obesity‐related parameters and hepatic lipid metabolism under HFHS diet conditions, with a particular focus on lipid metabolism‐ and fibrosis‐related gene expression. Collectively, our results showed that MC supplementation was associated with lower body weight gain and alterations in the expression of key lipogenic transcription factors and genes (*SREBP-1c*, *C/EBPα*, *PPARγ*, *ACC*, *FAS*, and *SCD1*) and fibrosis‐related genes (*Col1α1* and *αSMA*) in HFHS‐fed mice. In an HFHS diet‐induced obesity model, MC supplementation was associated with lower hepatic lipid accumulation and adipocyte size, together with changes in genes involved in the lipid metabolism. These findings suggest that MC may modulate lipid metabolism‐related responses under HFHS diet conditions.

Given the increasing prevalence of high‐fat, high‐sugar dietary patterns associated with metabolic disorders, these findings highlight the potential of MC as a functional food ingredient for managing obesity‐associated metabolic alterations.

## Author Contributions

Conceptualization, resources, writing – review and editing, supervision, project administration: Hyocheol Bae. Methodology, software, validation, formal analysis, data curation, writing – original draft, visualization: Hyun‐Min Woo and Se‐Yeong Park. Investigation: Hyocheol Bae, Yong‐Mu Kim, Hyun‐Min Woo, and Se‐Yeong Park.

## Funding

This research was supported by the World Class 300 Project (Project Number RS‐2023‐KI002736) funded by the Ministry of Trade, Industry and Energy (MOTIE, Korea), the Korea Institute for Advancement of Technology (KIAT), and the National Research Foundation of Korea (NRF) (Grant 2022R1C1C1007067).

## Disclosure

All authors have read and agreed to the published version of the manuscript.

## Ethics Statement

All animal experiments were approved by the Institutional Animal Care and Use Committee of Kyung Hee University (KHGASP‐24‐690), and were conducted in accordance with the relevant guidelines and regulations.

## Conflicts of Interest

The authors declare no conflicts of interest.

## Supporting Information

Additional supporting information can be found online in the Supporting Information section.

## Supporting information


**Supporting Information** Figure S1: Amino acid chromatograms from LA8080 analysis. (A) GABA standard solution (STD trace). (B) MC (test solution) showing GABA peak identification by retention time comparison (overlay or side‐by‐side; manufacturer’s raw data). Figure S2: Representative immunohistochemical staining of PPARγ and C/EBPα in liver tissue and PPARγ in adipose tissue. Representative images of each experimental group and their corresponding IgG‐negative controls are shown. Table S1: Body weight gain, total food intake, and food efficiency ratio (FER) in the experimental groups during the 12‐week supplementation period.

## Data Availability

The data that support the findings of this study are available from the corresponding author upon reasonable request.

## References

[1] Okunogbe A. , Nugent R. , Spencer G. , Powis J. , Ralston J. , and Wilding J. , Economic Impacts of Overweight and Obesity: Current and Future Estimates for 161 Countries, BMJ Global Health. (2022) 7, no. 9, 10.1136/bmjgh-2022-009773. PMC9494015 36130777

[2] Jung R. T. , Obesity as a Disease, British Medical Bulletin. (1997) 53, no. 2, 307–321, 10.1093/oxfordjournals.bmb.a011615. 9246838

[3] Johnson R. J. , Segal M. S. , and Sautin Y. , et al.Potential Role of Sugar (Fructose) in the Epidemic of Hypertension, Obesity and the Metabolic Syndrome, Diabetes, Kidney Disease, and Cardiovascular Disease, The American Journal of Clinical Nutrition. (2007) 86, no. 4, 899–906, 10.1093/ajcn/86.4.899. 17921363

[4] Stanhope K. L. , Sugar Consumption, Metabolic Disease and Obesity: The State of the Controversy, Critical Reviews in Clinical Laboratory Sciences. (2016) 53, no. 1, 52–67, 10.3109/10408363.2015.1084990. PMC4822166 26376619

[5] Bray G. A. , Kim K.-K. , Wilding J. P. , and Federation W. O. , Obesity: A Chronic Relapsing Progressive Disease Process. A Position Statement of the World Obesity Federation, Obesity Reviews. (2017) 18, no. 7, 715–723, 10.1111/obr.12551. 28489290

[6] Frayn K. , Adipose Tissue as a Buffer for Daily Lipid Flux, Diabetologia. (2002) 45, no. 9, 1201–1210, 10.1007/s00125-002-0873-y. 12242452

[7] Binns C. W. , Lee M. K. , and Lee A. H. , Problems and Prospects: Public Health Regulation of Dietary Supplements, Annual Review of Public Health. (2018) 39, no. 1, 403–420, 10.1146/annurev-publhealth-040617-013638. 29272167

[8] Grover J. K. and Yadav S. P. , Pharmacological Actions and Potential Uses of *Momordica charantia*: A Review, Journal of Ethnopharmacology. (2004) 93, no. 1, 123–132, 10.1016/j.jep.2004.03.035. 15182917

[9] Raman A. and Lau C. , Anti-Diabetic Properties and Phytochemistry of *Momordica charantia* L. (Cucurbitaceae), Phytomedicine. (1996) 2, no. 4, 349–362, 10.1016/S0944-7113(96)80080-8. 23194773

[10] Virdi J. , Sivakami S. , Shahani S. , Suthar A. C. , Banavalikar M. M. , and Biyani M. K. , Antihyperglycemic Effects of Three Extracts From, *Momordica charantia* , Journal of Ethnopharmacology. (2003) 88, no. 1, 107–111, 10.1016/S0378-8741(03)00184-3. 12902059

[11] Nerurkar P. V. , Johns L. M. , and Buesa L. M. , et al. *Momordica charantia* (Bitter Melon) Attenuates High-Fat Diet-Associated Oxidative Stress and Neuroinflammation, Journal of Neuroinflammation. (2011) 8, no. 1, 10.1186/1742-2094-8-64, 64. PMC3129574 21639917

[12] Ahmed I. , Adeghate E. , Cummings E. , Sharma A. K. , and Singh J. , Beneficial Effects and Mechanism of Action of *Momordica charantia* Juice in the Treatment of Streptozotocin-Induced Diabetes Mellitus in Rat, Molecular and Cellular Biochemistry. (2004) 261, no. 1, 63–70, 10.1023/B:MCBI.0000028738.95518.90. 15362486

[13] Hafizur R. M. , Kabir N. , and Chishti S. , Modulation of Pancreatic β-Cells in Neonatally Streptozotocin-Induced Type 2 Diabetic Rats by the Ethanolic Extract of *Momordica charantia* Fruit Pulp, Natural Product Research. (2011) 25, no. 4, 353–367, 10.1080/14786411003766904. 21328131

[14] Softic S. , Cohen D. E. , and Kahn C. R. , Role of Dietary Fructose and Hepatic De Novo Lipogenesis in Fatty Liver Disease, Digestive Diseases and Sciences. (2016) 61, no. 5, 1282–1293, 10.1007/s10620-016-4054-0. PMC4838515 26856717

[15] Moslehi A. and Hamidi-Zad Z. , Role of SREBPs in Liver Diseases: A Mini-Review, Journal of Clinical and Translational Hepatology. (2018) 6, no. 3, 10.14218/JCTH.2017.00061, 332. PMC6160306 30271747

[16] Nagai Y. , Nishio Y. , Nakamura T. , Maegawa H. , Kikkawa R. , and Kashiwagi A. , Amelioration of High Fructose-Induced Metabolic Derangements by Activation of PPARα, American Journal of Physiology-Endocrinology and Metabolism. (2002) 282, no. 5, E1180–E1190, 10.1152/ajpendo.00471.2001. 11934685

[17] Li N. , Li X. , and Ding Y. , et al.SREBP Regulation of Lipid Metabolism in Liver Disease, and Therapeutic Strategies, Biomedicines. (2023) 11, no. 12, 10.3390/biomedicines11123280, 3280. PMC10740981 38137501

[18] Choi Y.-J. , Shin H.-S. , and Choi H. S. , et al.Uric Acid Induces Fat Accumulation via Generation of Endoplasmic Reticulum Stress and SREBP-1c Activation in Hepatocytes, Laboratory Investigation. (2014) 94, no. 10, 1114–1125, 10.1038/labinvest.2014.98. 25111690

[19] Miyazaki M. , Dobrzyn A. , and Man W. C. , et al.Stearoyl-CoA desaturase 1 Gene Expression Is Necessary for Fructose-Mediated Induction of Lipogenic Gene Expression by Sterol Regulatory Element-Binding Protein-1c-Dependent and-Independent Mechanisms, Journal of Biological Chemistry. (2004) 279, no. 24, 25164–25171, 10.1074/jbc.M402781200. 15066988

[20] Sun Q. , Xing X. , and Wang H. , et al.SCD1 Is the Critical Signaling Hub to Mediate Metabolic Diseases: Mechanism and the Development of Its Inhibitors, Biomedicine & Pharmacotherapy. (2024) 170, 10.1016/j.biopha.2023.115586, 115586. 38042113

[21] Venetos N. M. , Stomberski C. T. , Qian Z. , Premont R. T. , and Stamler J. S. , Activation of Hepatic Acetyl-CoA Carboxylase by S-Nitrosylation in Response to Diet, Journal of Lipid Research. (2024) 65, no. 5, 10.1016/j.jlr.2024.100542, 100542. PMC11126798 38641009

[22] Jensen-Urstad A. P. L. and Semenkovich C. F. , Fatty Acid Synthase and Liver Triglyceride Metabolism: Housekeeper or Messenger?, Biochimica et Biophysica Acta (BBA) - Molecular and Cell Biology of Lipids. (2012) 1821, no. 5, 747–753, 10.1016/j.bbalip.2011.09.017. PMC3288544 22009142

[23] Zhou J. , Febbraio M. , and Wada T. , et al.Hepatic Fatty Acid Transporter Cd36 Is a Common Target of LXR, PXR, and PPARγ in Promoting Steatosis, Gastroenterology. (2008) 134, no. 2, 556–567.e1, 10.1053/j.gastro.2007.11.037. 18242221

[24] Christodoulides C. , Lagathu C. , Sethi J. K. , and Vidal-Puig A. , Adipogenesis and WNT Signalling, Trends in Endocrinology & Metabolism. (2009) 20, no. 1, 16–24, 10.1016/j.tem.2008.09.002. PMC4304002 19008118

[25] Tontonoz P. and Spiegelman B. M. , Fat and Beyond: The Diverse Biology of PPARγ, Annual Review of Biochemistry. (2008) 77, no. 1, 289–312, 10.1146/annurev.biochem.77.061307.091829. 18518822

[26] Matsusue K. , Haluzik M. , and Lambert G. , et al.Liver-Specific Disruption of PPARγ in Leptin-Deficient Mice Improves Fatty Liver but Aggravates Diabetic Phenotypes, Journal of Clinical Investigation. (2003) 111, no. 5, 737–747, 10.1172/JCI200317223. PMC151902 12618528

[27] Boelsterli U. A. and Bedoucha M. , Toxicological Consequences of Altered Peroxisome Proliferator-Activated Receptor γ (PPARγ) Expression in the Liver: Insights From Models of Obesity and Type 2 Diabetes, Biochemical Pharmacology. (2002) 63, no. 1, 1–10, 10.1016/S0006-2952(01)00817-6. 11754868

[28] Lee Y. K. , Park J. E. , Lee M. , and Hardwick J. P. , Hepatic Lipid Homeostasis by Peroxisome Proliferator-Activated Receptor Gamma 2, Liver Research. (2018) 2, no. 4, 209–215, 10.1016/j.livres.2018.12.001. PMC6594548 31245168

[29] Ahmadian M. , Suh J. M. , and Hah N. , et al.PPARγ Signaling and Metabolism: The Good, the Bad and the Future, Nature Medicine. (2013) 19, no. 5, 557–566, 10.1038/nm.3159. PMC3870016 23652116

[30] Chiang D. J. , Pritchard M. T. , and Nagy L. E. , Obesity, Diabetes Mellitus, and Liver Fibrosis, American Journal of Physiology-Gastrointestinal and Liver Physiology. (2011) 300, no. 5, G697–G702, 10.1152/ajpgi.00426.2010. PMC3094133 21350183

[31] Al Zarzour R. H. , Alshawsh M. A. , and Asif M. , et al.Adipocytokine Regulation and Antiangiogenic Activity Underlie the Molecular Mechanisms of Therapeutic Effects of *Phyllanthus niruri* Against Non-Alcoholic Fatty Liver Disease, Nutrients. (2018) 10, no. 8, 10.3390/nu10081057, 1057. PMC6115813 30096951

[32] Pessoa J. and Teixeira J. , Cytoskeleton Alterations in Non-Alcoholic Fatty Liver Disease, Metabolism. (2022) 128, 10.1016/j.metabol.2021.155115, 155115. 34974078

[33] Friedman S. L. , Mechanisms of Hepatic Fibrogenesis, Gastroenterology. (2008) 134, no. 6, 1655–1669, 10.1053/j.gastro.2008.03.003. PMC2888539 18471545

[34] Henderson N. C. and Iredale J. P. , Liver Fibrosis: Cellular Mechanisms of Progression and Resolution, Clinical Science. (2007) 112, no. 5, 265–280, 10.1042/CS20060242. 17261089

[35] Tsuchida T. and Friedman S. L. , Mechanisms of Hepatic Stellate Cell Activation, Nature Reviews Gastroenterology & Hepatology. (2017) 14, no. 7, 397–411, 10.1038/nrgastro.2017.38. 28487545

[36] Poret J. M. , Souza-Smith F. , and Marcell S. J. , et al.High Fat Diet Consumption Differentially Affects Adipose Tissue Inflammation and Adipocyte Size in Obesity-Prone and Obesity-Resistant Rats, International Journal of Obesity. (2018) 42, no. 3, 535–541, 10.1038/ijo.2017.280. PMC5876080 29151595

[37] Reynisdottir S. , Ellerfeldt K. , Wahrenberg H. , Lithell H. , and Arner P. , Multiple Lipolysis Defects in the Insulin Resistance (Metabolic) Syndrome, Journal of Clinical Investigation. (1994) 93, no. 6, 2590–2599, 10.1172/JCI117271. PMC294492 8200997

[38] Le Lay S. , Krief S. , and Farnier C. , et al.Cholesterol, a Cell Size-dependent Signal That Regulates Glucose Metabolism and Gene Expression in Adipocytes, Journal of Biological Chemistry. (2001) 276, no. 20, 16904–16910, 10.1074/jbc.M010955200. 11278795

[39] Suganami T. and Ogawa Y. , Adipose Tissue Macrophages: Their Role in Adipose Tissue Remodeling, Journal of Leukocyte Biology. (2010) 88, no. 1, 33–39, 10.1189/jlb.0210072. 20360405

[40] Bensinger S. J. and Tontonoz P. , Integration of Metabolism and Inflammation by Lipid-Activated Nuclear Receptors, Nature. (2008) 454, no. 7203, 470–477, 10.1038/nature07202. 18650918

[41] Horton J. D. , Goldstein J. L. , and Brown M. S. , SREBPs: Activators of the Complete Program of Cholesterol and Fatty Acid Synthesis in the Liver, Journal of Clinical Investigation. (2002) 109, no. 9, 1125–1131, 10.1172/JCI0215593. PMC150968 11994399

[42] Lee K. W. , Shim J. M. , Yao Z. , Kim J. A. , and Kim J. H. , Properties of Kimchi Fermented With GABA-Producing Lactic Acid Bacteria as a Starter, Journal of Microbiology and Biotechnology. (2018) 28, no. 4, 534–541. 10.4014/jmb.1709.09011 29724081

[43] Kim Y. K. , Xu H. , Park N. I. , Boo H. O. , Lee S. Y. , and Park S. U. , Amino Acid and GABA Content in Different Cultivars of *Momordica charantia* L, Journal of Medicinal Plants Research. (2009) 3, no. 11, 897–U1402.

[44] Chow J. D. , Lawrence R. T. , and Healy M. E. , et al.Genetic Inhibition of Hepatic Acetyl-CoA Carboxylase Activity Increases Liver Fat and Alters Global Protein Acetylation, Molecular Metabolism. (2014) 3, no. 4, 419–431, 10.1016/j.molmet.2014.02.004. PMC4060285 24944901

