# Supplementary material for: Momordica charantia Attenuates Hepatic Steatosis in High‐Fat, High‐Sucrose Diet‐Induced Obese Mice
Source: Anal Cell Pathol (Amst). 2026 Oct 26;2026:2235628. doi: 10.1155/ancp/2235628 (PMC13700938; doi:10.1155/ancp/2235628)
Supplement: Supplementary file 1 [file ANCP-2026-2235628-s001.zip › Supplementary_Table_1.docx]

**Supplementary Table 1.** Body weight gain, total food intake, and food efficiency ratio (FER) in the experimental groups during the 12-week supplementation period.

| Group | Body weight gain (g) | Total food intake (g) | FER |
| --- | --- | --- | --- |
| NC | 2.34 | 2104.1667 | 0.0067 |
| HFHS | 10.84 | 2053.1667 | 0.0317 |
| CQ | 11.73 | 2119.8750 | 0.0332 |
| MC 125 | 12.66 | 2074.8750 | 0.0366 |
| MC 250 | 11.45 | 2193.1250 | 0.0313 |
